# High Expression of E2F4 Is an Adverse Prognostic Factor and Related to Immune Infiltration in Oral Squamous Cell Carcinoma

**DOI:** 10.1155/2022/4731364

**Published:** 2022-12-15

**Authors:** Yicai Zheng, He Fei

**Affiliations:** ^1^Department of Stomatology, The Fifth People's Hospital of Shanghai, Fudan University, Shanghai 200240, China; ^2^Department of Gynecology, The Fifth People's Hospital of Shanghai, Fudan University, Shanghai 200240, China

## Abstract

**Background:**

We aimed to evaluate the prognostic value of E2F4 expression in oral squamous cell carcinoma (OSCC) and clarify its influence on immune cell infiltration and biological functions.

**Methods:**

The Cancer Genome Atlas (TCGA) database, the STRING database, and related online tools as well as single-sample gene set enrichment analysis (ssGSEA) were used for the analyses in our study.

**Results:**

The E2F4 expression was elevated in OSCC tumor tissue compared with paracancerous tissues. The high expression of E2F4 was closely related to the poorer overall survival, disease-free survival, and progression-free interval of OSCC. In addition, pathway enrichment analyses revealed that the top 49 genes most closely related to E2F4 were strongly associated with the cell cycle. E2F4-related proteins were closely related to the following KEGG pathways: cell cycle, cellular senescence, PI3K-Akt signaling pathway, Wnt signaling pathway, and notch signaling pathway. It was also demonstrated that the E2F4 expression was negatively correlated with immune purity and strongly related to immune cell infiltration in OSCC.

**Conclusions:**

E2F4 can be used as a novel biomarker for the diagnosis and prognosis of OSCC.

## 1. Introduction

Oral cancer is overall the eighth most common malignancy [1]. Oral squamous cell carcinoma (OSCC) accounts for over 95% of oral cancers, and more than 500,000 individuals are diagnosed with OSCC each year in the world [2]. Recent years have witnessed remarkable improvements in the diagnosis and treatment of OSCC. However, due to insufficient early detection, the 5-year survival rate of OSCC patients is still less than 50% [3]. Therefore, identification of effective biomarkers for OSCC and a better understanding of the molecular pathology of OSCC are critical to improving overall survival.

The E2F family was first identified in 1987 as transcription factors required to activate the E2 adenovirus promoter [4]. Nine members of the family are located on different chromosomes and subdivided into activators (E2F1, E2F2, and E2F3a) and repressors (E2F3b, E2F4, E2F5, E2F6, E2F7, and E2F8) based on detail functions [5]. Abnormal expression of some E2F family transcription factors has been found in numerous human malignancies including ovarian [6], gastric [7], and colorectal cancer [8]. E2Fs are also supposed to have complex and distinct roles in OSCC. The E2F1 expression was upregulated in OSCC tumor tissues compared to paracancerous tissues [9]. SNHG12/miR-326/E2F1 feedback loop facilitated OSCC development [10]. These results indicate that E2F1 plays an oncogenic role in OSCC. E2F7 was highly expressed in OSCC tissues. Besides, high expression of E2F7 predicted worse prognosis in OSCC patients. Gain and loss of function assays displayed that E2F7 performs a carcinogenic role in OSCC [11]. In contrary, little is known about how E2F4 was regulated in OSCC. Moreover, the exact underlying mechanism of E2F4 as an oncogene needs further investigation.

Immune dysfunction has been shown to be closely related to tumorigenesis and progression. Tumor-infiltrating lymphocytes (TILs) are considered to be the host immune response to tumor cells [12]. Different types of TILs have different effects on tumor growth, recurrence, and metastasis [13]. The presence of TILs is significantly associated with the prognosis of various solid tumors [14–16], including OSCC [17]. OSCC is often ulcerated with massive lymphocytic infiltration. It is important to improve the understanding of tumor immune microenvironment of OSCC, including the correlation between tumor-related factors and TILs, as to better understand the pathogenesis of OSCC. A series of evidence has shown that E2F4 is significantly correlated with TILs [18]. In mammals, E2F4 is a key member of transcription factors involved in innate immune responses, such as the expression of Toll-like receptor 8 and STAT1 pathway [19]. The transcription levels of E2Fs are closely correlated with various levels of immune infiltration in pancreatic adenocarcinoma [20]. However, the correlation between E2Fs and TILs in head and neck squamous cell carcinomas remains unclear.

The purpose of this study was to evaluate the prognostic value of E2F4 in OSCC based on data obtained from The Cancer Genome Atlas (TCGA) database. To gain further insight into the potential functions, the biological pathways involved in OSCC pathogenesis-related E2F4 regulatory network and gene set enrichment analysis were performed based on data obtained from the STRING database. Moreover, the relationship of E2F4 with TILs was analysed using single-sample gene set enrichment analysis (ssGSEA).

## 2. Materials and Methods

### 2.1. TCGA Data Download

RNA seq data in HT Seq-FPKM (fragments per kilobase per million) format from the Head and Neck Squamous Cell Carcinoma (HNSC) project were downloaded from TCGA database (https://portal.gdc.cancer.gov/), retaining samples belonging to oral cancer sites (alveolar ridge, base of tongue, buccal mucosa, floor of mouth, hard palate, oral cavity, and oral tongue) and excluding nonoral cancer sites (hypopharynx, larynx, lip, oropharynx, and tonsil). A total of 329 OSCC tissues and 32 paracancerous tissues were included.

### 2.2. Expression Level and Prognosis of E2F mRNA in OSCC

The expression of E2Fs was normalized to transcripts per million reads (TPM) value before further analysis. The Shapiro-Wilk normality test is used to test the normality distribution of samples. The difference in mRNA levels between two groups were analyzed with Wilcoxon rank sum test. The Kruskal-Wallis test was performed to evaluate the difference among the three groups, and the Bonferroni method was chosen for the correction of *P* value. Combined with clinical characteristics, the Kaplan-Meier method was applied to analyse the correlation between E2F family genes and survival prognosis of OSCC and to investigate the differences and significance of the expression of E2Fs in different age, gender, clinical stage, and histologic grade groups. Based on these results, core important genes were screened and uniformly processed TCGA and GTEx pan-cancer RNA seq data were obtained from the UCSC Xena website (https://xena.ucsc.edu/) for pan-cancer expression differential analysis [21], including adrenocortical carcinoma (ACC), bladder urothelial carcinoma (BLCA), breast invasive carcinoma (BRCA), cervical squamous cell carcinoma and endocervical adenocarcinoma (CESC), cholangiocarcinoma (CHOL), colon adenocarcinoma (COAD), lymphoid neoplasm diffuse large B cell lymphoma (DLBC), esophageal carcinoma (ESCA), glioblastoma multiforme (GBM), head and neck squamous cell carcinoma (HNSC), kidney chromophobe (KICH), kidney renal clear cell carcinoma (KIRC), kidney renal papillary cell carcinoma (KIRP), acute myeloid leukemia (LAML), brain lower grade glioma (LGG), liver hepatocellular carcinoma (LIHC), lung adenocarcinoma (LUAD), lung squamous cell carcinoma (LUSC), mesothelioma (MESO), ovarian serous cystadenocarcinoma (OV), pancreatic adenocarcinoma (PAAD), pheochromocytoma and paraganglioma (PCPG), prostate adenocarcinoma (PRAD), rectum adenocarcinoma (READ), sarcoma (SARC), skin cutaneous melanoma (SKCM), stomach adenocarcinoma (STAD), testicular germ cell tumors (TGCT), thyroid carcinoma (THCA), thymoma (THYM), uterine corpus endometrial carcinoma (UCEC), uterine carcinosarcoma (UCS), and uveal melanoma (UVM). *P* < 0.05 was considered statistically significant.

### 2.3. Interacting Genes of E2Fs

The STRING (https://string-db.org/, version: 11.5) is an online database search for known protein interactions [22]. Select the “Single Protein by Name” section to search for genes that are closely related to E2Fs and test for functional and pathway enrichment analyses of these interacting genes. The confidence score was set at >0.9 for the PPI analysis. Then, the protein interaction data was imported into the Cytoscape software (version 3.9.0) to obtain a network map [23]. After obtaining the interacting genes of E2Fs, we then performed GO and KEGG pathway analyses using R package “clusterProfiler” with enrichment *P* < 0.05.

### 2.4. Association between E2Fs and Prognosis of Patients with OSCC

After obtaining absolute TPM value of E2Fs of each OSCC sample, OSCC case was divided into two groups with median value of TPM (5.87) as the cut-off value. The Wilcoxon rank sum tests were used to test both groups. Next, univariate and multivariate Cox proportional risk regression analyses were performed using the “survival” package of the R software, including variables such as E2Fs expression, gender, age, clinical stage, and histologic grade. *P* < 0.05 was considered statistically significant.

### 2.5. Tumor Microenvironment and Immune Function Analysis

The tumor microenvironment (TME) is an environment composed of tumor cells, extracellular matrix (ECM), tumor-associated stromal cells, tumor-associated immune cells, and signaling molecules [24]. StromalScores, ImmuneScore, and ESTIMATEScores were assessment methods to quantify the tumor microenvironment [25]. The “estimate” package was used to explore the differences in StromalScore, ImmuneScore, and ESTIMATEScores between the two groups under the high- and low-expression groupings of E2Fs. In addition, their immune function was assessed by quantifying the tumor-infiltrating immune cells between the two groups by ssGSEA algorithm [26]. The ssGSEA was performed in R package GSVA [27]. The difference was considered statistically significant at *P* < 0.05.

### 2.6. Immune Cell Infiltration Analysis

TIMER is a public resource platform that provides a comprehensive analysis of immune cell infiltration in various cancers [28]. The abundance of different types of infiltrating immune cells (CD4^+^ T cells, CD8^+^ T cells, B cells, dendritic cells, macrophages, and neutrophils) in OSCC samples was obtained, and the correlation between the expression of E2Fs and these immune cells was explored by Spearman. Moreover, the correlation between E2F expression and different immune cells (aDC (activated DC), B cells, CD8^+^ T cells, cytotoxic cells, DCs, eosinophils, iDC (immature DC), macrophages, mast cells, neutrophils, NK CD56bright cells, NK CD56dim cells, NK cells, pDC (plasmacytoid DC), T cells, T helper cells, Tcm (T central memory), Tem (T effector memory), Tfh (T follicular helper), Tgd (T gamma delta), Th1 cells, Th17 cells, Th2 cells, and Treg) was further explored based on the ssGSEA algorithm. *P* < 0.05 was statistically significant.

## 3. Results

### 3.1. Expression and Prognosis of the E2F Family Genes in OSCC

**Figure 1:**
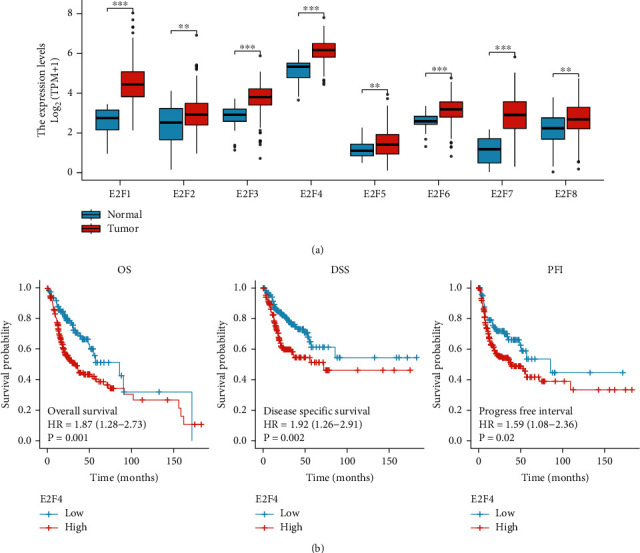
Expression level of E2F family and prognostic analysis of E2F4 in OSCC. (a) The expression of the E2F family was significantly upregulated in OSCC tissues. (b) OSCC patients with high expression of E2F4 had poor OS, DSS, and PFI. OSCC: oral squamous cell carcinoma; ^∗^*P* value < 0.05; ^∗∗^*P* value < 0.01; ^∗∗∗^*P* value < 0.001.

**Figure 2:**
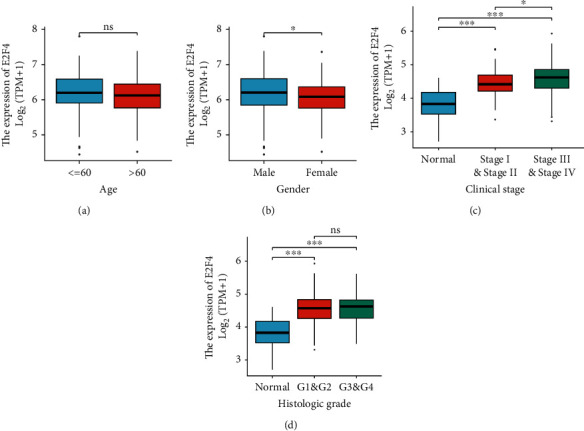
Correlation of E2F4 expression with clinical characteristics of patients with OSCC. E2F4 expression was statistically significant in Gender (B), Clinical stage (c) and Histologic grade (d) subgroups, but not in Age (a) subgroup. ^∗^*P* value < 0.05; ^∗∗^*P* value < 0.01; ^∗∗∗^*P* value < 0.001.

Analysis of the TCGA database revealed that the expression levels of all E2F family members were significantly upregulated in OSCC tissues compared to paracancerous tissues (Figure 1(a)). To further assess the prognostic value of the E2F family in OSCC, the correlation between the expression of E2Fs and overall survival (OS), disease-free survival (DSS), and progression-free interval (PFI) was analysed by the Kaplan-Meier method. The results showed that only E2F4 was significantly related to OS, DSS, and PFI. The high expression of E2F4 was closely related to the poorer OS (*P* = 0.001), DSS (*P* = 0.002), and PFI (*P* = 0.02) of OSCC (Figure 1(b)). In addition, the corresponding OSCC subgroups are divided according to different clinical characteristic parameters, and it was found that the expression of E2F4 was significantly increased in male patients, and as the clinical stage and histologic grade increased, the expression level of E2F4 increased in turn (Figures 2(b)–2(d)). The above results suggested that E2F4 may be involved in the tumor process of OSCC and could be used as a potential prognostic marker for OSCC. Interestingly, E2F4 was not only upregulated in OSCC tissues but also significantly upregulated in CHOL, COAD, DLBC, ESCA, GBM, KIRC, LGG, PAAD, READ, SKCM, STAD, THYM, and UCS tissues (Supplementary Figure 1).

### 3.2. Prognostic Value of E2F4 in OSCC

**Figure 3:**
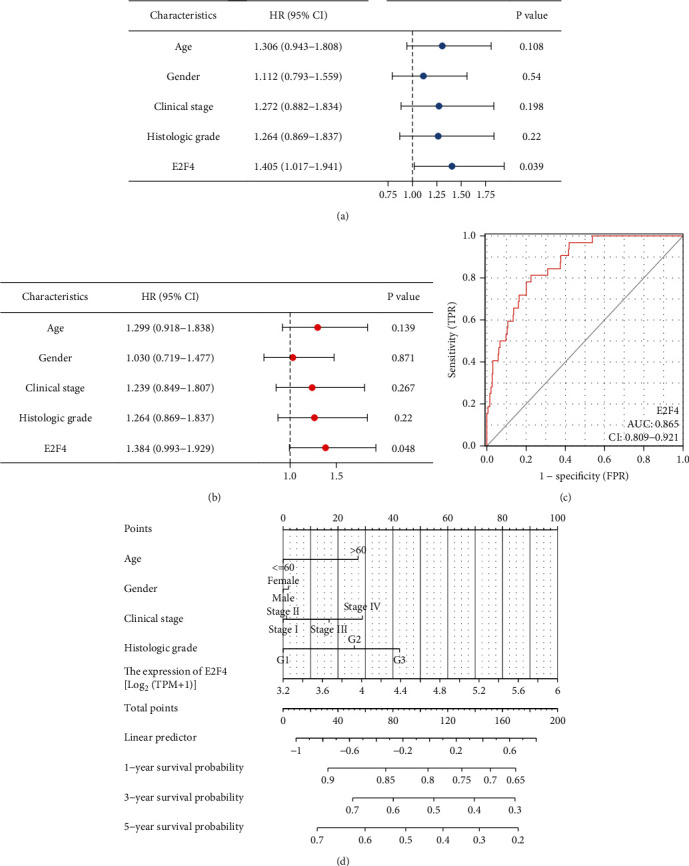
Analysis of the predictive prognostic ability of E2F4 in OSCC. Univariate (a) and multivariate (b) Cox proportional risk regression analyses of E2F4. (c) The AUC values of the E2F4. (d) A nomogram for both clinic-pathological factors and E2F4. OSCC: oral squamous cell carcinoma; AUC: area under curve.

We further demonstrated the association between E2F4 and different clinical characteristics of OSCC and found that E2F4 expression was significantly associated with gender and clinical stage of OSCC (Table 1). To further confirm the clinical diagnostic value of E2F4, we performed univariate and multivariate Cox regression analyses on E2F4 and clinical characteristics in OSCC (Table 2 and Figures 3(a) and 3(b)). The E2F4 expression levels could be an independent prognostic factor for OSCC. Furthermore, E2F4 had good accuracy (AUC = 0.865) in predicting OSCC prognosis (Figure 3(c)). Finally, a nomogram was constructed to predict the 1-, 3-, and 5-year survival rates of OSCC patients (Figure 3(d)).

### 3.3. Functional and Pathway Enrichment Analyses of E2F4-Related Proteins

**Figure 4:**
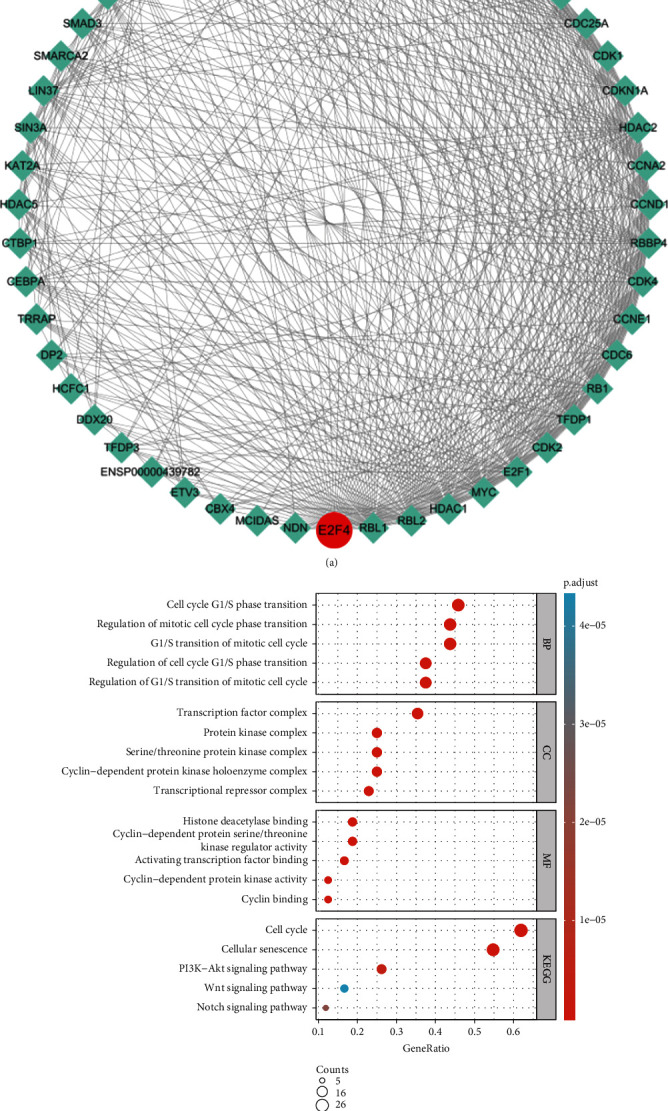
Functional and pathway enrichment analyses of E2F4-related genes. (a) PPI network of the 49 interacting genes of E2F4. (b) GO functional enrichment analysis of the relevant biological process, cellular components, and molecular functions of interacting genes of E2F4; KEGG pathway analysis of the relevant signal pathways of interacting genes of E2F4. PPI: protein-protein interaction; GO: Gene Ontology; BP: biological process; CC: cellular component; MF: molecular functions; KEGG: Kyoto Encyclopedia of Genes and Genomes.

First, the 49 genes most closely related to E2F4 were identified through the STRING database, and a protein interaction network was constructed using Cytoscape software (Figure 4(a)). Subsequent functional and pathway enrichment analyses showed that GO functional enrichment analysis mainly involves cell cycle G1/S phase transition, G1/S transition of mitotic cell cycle, regulation of G1/S transition of mitotic cell cycle, regulation of cell cycle G1/S phase transition, regulation of mitotic cell cycle phase transition, and other links. In terms of cellular components, it is mainly highly enriched in the following cyclin-dependent protein kinase, holoenzyme complex, serine/threonine protein kinase complex, transcription factor complex, protein kinase complex, transcriptional repressor complex, and other components. In terms of molecular functions, cyclin-dependent protein serine/threonine kinase regulator activity, histone deacetylase binding, activating transcription factor binding, cyclin binding, and cyclin-dependent protein kinase activity are the most enriched. In addition, E2F4-related proteins were closely related to the following KEGG pathways: cell cycle, cellular senescence, PI3K-Akt signaling pathway, Wnt signaling pathway, and Notch signaling pathway (Figure 4(b)). The results were summarized in Supplementary Table 1.

### 3.4. E2F4 Was Closely Associated with the TME and Immune Cell Infiltration in OSCC

**Figure 5:**
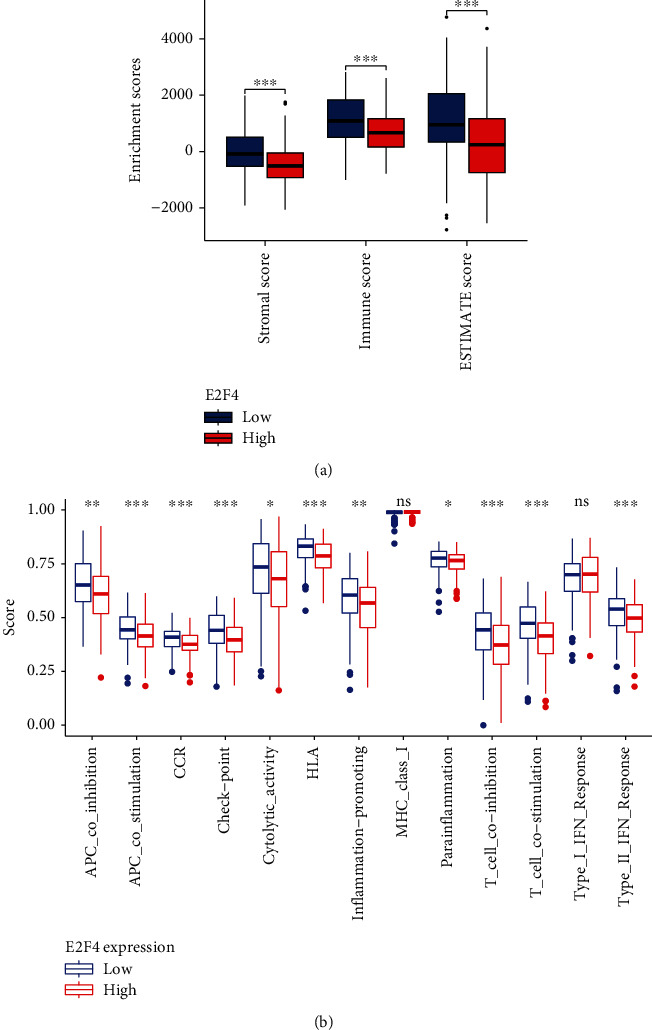
Correlation of E2F4 with tumor microenvironment and immune function. (a) OSCC patients with high E2F4 expression had low StromalScore, ImmuneScore, and ESTIMATEScore. (b) Exploring immune-related functions between high and low E2F4 expression subgroups by the ssGSEA algorithm. ssGSEA: single-sample GSEA. ^∗^*P* value < 0.05; ^∗∗^*P* value < 0.01; ^∗∗∗^*P* value < 0.001.

**Figure 6:**
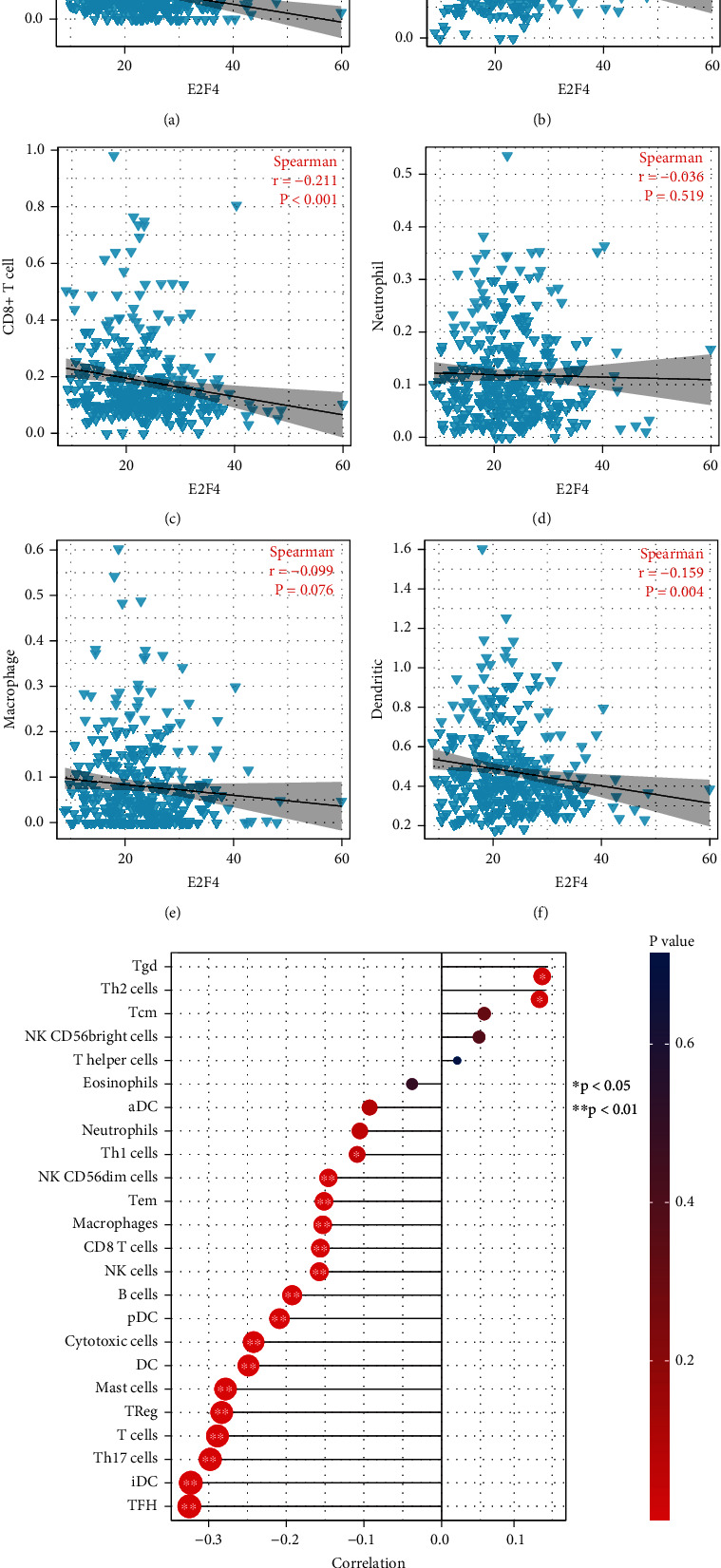
Correlation of E2F4 with tumor immune cell infiltration (TIMER and ssGSEA). (a–f) E2F4 expression levels were negatively and significantly correlated with the abundance of B cells, CD4^+^ T cells, CD8^+^ T cells, neutrophil, macrophage, and dendritic cells (Timer). (g) E2F4 expression levels were significantly and positively correlated with the abundance of Th2 cells, Tcm, and Tgd cells and negatively correlated with the abundance of NK CD56dim cells, CD8 T cells, DC, B cells, Treg, mast cells, pDC, iDC, TFH, cytotoxic cells, T cells, and Th17 cells (ssGSEA). ssGSEA: single-sample GSEA. ^∗^*P* -value < 0.05; ^∗∗^*P* value < 0.01; ^∗∗∗^*P* value < 0.001.

Immune cell infiltration in tumors is an integral part of TME, and the estimate and ssGSEA algorithms were used to assess the proportion of each component of the TME and the correlation between tumor immune cell infiltration and E2F4. The results showed that patients in the high-expression group of E2F4 had a low StromalScore and ImmuneScore (Figure 5(a)). In terms of immune function, APC coinhibition, APC costimulation, CCR, check point, cytolytic activity, HLA, inflammation promoting, parainflammation, T cell coinhibition, T cell costimulation, and type II IFN response differed significantly between the high- and low-expression groups (Figure 5(b)). We selected TIMER to clarify the Spearman correlation between E2F4 and immune infiltrating lymphocytes in OSCC, which showed a strong negative correlation between E2F4 and immune infiltrating lymphocytes, including B cells, CD8^+^ T cells, and dendritic cells (*P* < 0.05) (Figures 6(a)–6(f)). Based on the ssGSEA algorithm to assess the degree of tumor immune infiltration, the results also showed a significant positive correlation between E2F4 expression levels and the abundance of Th2 cells, Tcm, and Tgd cells and a negative correlation with the abundance of NK CD56dim cells, CD8^+^ T cells, DC, T cells, B cells, Treg, mast cells, pDC, iDC, TFH, cytotoxic cells, and Th17 cells (Figure 6(g)).

## 4. Discussion

In this study, we observed that the E2F4 expression was upregulated in OSCC tissues compared with paracancerous tissues, and the Kaplan-Meier analyses suggested that E2F4 might be a potential prognostic factor for OSCC. We further provided a detailed picture of E2F4 expression in OSCC in relation to clinical features, pathway crosstalk, and immune cell infiltration.

E2F4 is a critical molecule in the RB/E2F pathway. Under physiological effects, E2F4 is the major repressor of cell cycle progression, which can prevent uncontrolled proliferation [29]. However, deletion of E2F4 in circulating stem and progenitor cell populations in multiple tissue types actually reduces proliferation and DNA replication, such as intestinal tissue [30], blood cells [31], and the developing epidermis [32]. In cancer, E2F4 appears to act primarily as an oncogene, which is more consistent with its role in promoting proliferation rather than its classic inhibitory effect on the cell cycle. E2F4 was highly expressed in hepatocellular carcinoma [18] as well as breast cancers [33] and strongly correlates with poor prognosis. In this study, we observed that E2F4 expression was upregulated in OSCC, and the high expression of E2F4 was closely related to the poorer OS, DSS, and PFI of OSCC. These findings are consistent with the tumor-promoting effect of E2F4 shown in previous studies and suggest that E2F4 may serve as a new biomarker for the prognosis of OSCC.

TME plays an important role in the carcinogenesis of OSCC. Infiltration of immune cells has predicted improved prognosis in many different tumor types [34]. E2F4 expression was found to be significantly associated with immune cell infiltration among lymphoma [19], pancreatic adenocarcinoma [20], and hepatocellular carcinoma [18]. In pancreatic adenocarcinoma patients, as a tumor suppressor gene, E2F4 expressions were positively correlated with the infiltration of B cells, CD8^+^ T cells, macrophages, neutrophils, and dendritic cells [20]. However, in this study, E2F4 was revealed as an oncogene in OSCC, patients in the high-expression group of E2F4 had a low ImmuneScore, and the E2F4 expression was negatively correlated with immune infiltrating lymphocytes, including B cells, CD8^+^ T cells, and dendritic cells. Therefore, we conclude that E2F4 may promote OSCC progression by mediating immune-related pathways. Further experiments, such as the correlation between E2F4 expression and degree of immune infiltrating cells in OSCC by immunohistochemistry, can better verify the role of E2F4 in immune cell infiltration.

Taken together, these findings demonstrate that the upregulation of E2F4 may be a molecular marker that predicts the prognosis of OSCC. Reasonably, the potential mechanisms of E2F4 in immune environment regulation still require further validation.

## Figures and Tables

**Table 1:** Clinical characteristics of the OSCC.

Characteristic	Low expression of E2F4	High expression of E2F4	*P* value
*n*	164	165	
Age, *n* (%)			0.184
≤60	71	84	
>60	93	80	
Gender, *n* (%)			0.005
Female	63	39	
Male	101	126	
Clinical stage, *n* (%)			0.008
Stage I	9	2	
Stage II	51	31	
Stage III	31	36	
Stage IV	73	96	
Histologic grade, *n* (%)			0.075
G1	35	18	
G2	96	108	
G3	32	37	
G4	1	2	

**Table 2:** Univariate and multivariate Cox proportional risk regression analyses of E2F4.

Characteristics	Total (*n*)	Univariate analysis		Multivariate analysis	
		Hazard ratio (95% CI)	*P* value	Hazard ratio (95% CI)	*P* value
Age	327	1.306 (0.943-1.808)	0.108	1.299 (0.918-1.838)	0.139
≤60	155				
>60	172				
Gender	328	1.112 (0.793-1.559)	0.540	1.030 (0.719-1.477)	0.871
Male	226				
Female	102				
Clinical stage	318	1.272 (0.882-1.834)	0.198	1.239 (0.849-1.807)	0.267
Stage I-II	90				
Stage III-IV	228				
Histologic grade	320	1.264 (0.869-1.837)	0.220	1.214 (0.831-1.773)	0.315
G1-2	251				
G3-4	69				
E2F4	328	1.405 (1.017-1.941)	0.039	1.384 (0.993-1.929)	0.048
Low	164				
High	164				

## Data Availability

*Materials and Methods*: RNA seq data in HT Seq-FPKM (fragments per kilobase per million) format from the Head and Neck Squamous Cell Carcinoma (HNSC) project were downloaded from TCGA database (https://portal.gdc.cancer.gov/), retaining samples belonging to oral cancer sites (alveolar ridge, base of tongue, buccal mucosa, floor of mouth, hard palate, oral cavity, and oral tongue) and excluding nonoral cancer sites (hypopharynx, larynx, lip, oropharynx, and tonsil). The STRING (https://string-db.org/) is an online database search for known protein interactions. Select the “Single Protein by Name” section to search for genes that are closely related to E2Fs and test for functional and pathway enrichment analyses of these interacting genes.

## Supplementary Materials

Supplementary 1 Supplementary Figure 1: expression level of E2F4 in pan-cancer (GTEx database). ^∗^*P* value < 0.05; ^∗∗^*P* value < 0.01; ^∗∗∗^*P* value < 0.001.

Supplementary 2 Supplementary Table 1: functional enrichment analysis of 49 interacting genes of E2F4.
