# Supplementary figures and images for: High Expression of E2F4 Is an Adverse Prognostic Factor and Related to Immune Infiltration in Oral Squamous Cell Carcinoma
Source: Biomed Res Int. 2022 Dec 15;2022:4731364. doi: 10.1155/2022/4731364 (PMC9780755; doi:10.1155/2022/4731364)

The expression of E2F4

 $\text{Log}_2(\text{TPM}+1)$ 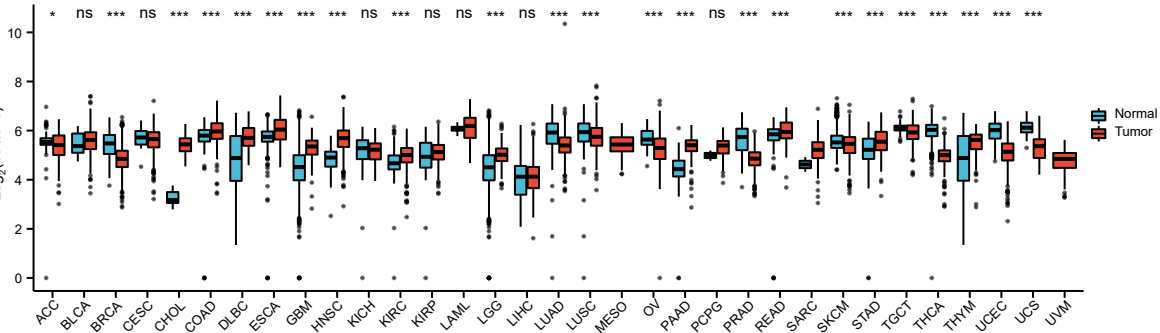

Supplement: Supplementary 1 — Supplementary Figure 1: expression level of E2F4 in pan-cancer (GTEx database). ∗P value < 0.05; ∗∗P value < 0.01; ∗∗∗P value < 0.001. [file 4731364.f1.pdf]
