# Supplementary material for: High Expression of E2F4 Is an Adverse Prognostic Factor and Related to Immune Infiltration in Oral Squamous Cell Carcinoma
Source: Biomed Res Int. 2022 Dec 15;2022:4731364. doi: 10.1155/2022/4731364 (PMC9780755; doi:10.1155/2022/4731364)
Supplement: Supplementary 2 — Supplementary Table 1: functional enrichment analysis of 49 interacting genes of E2F4. [file 4731364.f2.docx]

**Supplementary Table 1 Functional enrichment analysis of 49 interacting genes of E2F4**

| ONTOLOGY | ID | Description | GeneRatio | BgRatio | pvalue | p.adjust | qvalue |
| --- | --- | --- | --- | --- | --- | --- | --- |
| BP | GO:0044843 | cell cycle G1/S phase transition | 22/48 | 298/18670 | 2.54e-27 | 4.08e-24 | 2.48e-24 |
| BP | GO:0000082 | G1/S transition of mitotic cell cycle | 21/48 | 279/18670 | 3.36e-26 | 2.70e-23 | 1.64e-23 |
| BP | GO:2000045 | regulation of G1/S transition of mitotic cell cycle | 18/48 | 184/18670 | 1.86e-24 | 9.98e-22 | 6.06e-22 |
| BP | GO:1902806 | regulation of cell cycle G1/S phase transition | 18/48 | 202/18670 | 1.05e-23 | 4.23e-21 | 2.57e-21 |
| BP | GO:1901990 | regulation of mitotic cell cycle phase transition | 21/48 | 444/18670 | 6.15e-22 | 1.98e-19 | 1.20e-19 |
| CC | GO:0000307 | cyclin-dependent protein kinase holoenzyme complex | 12/48 | 42/19717 | 1.02e-22 | 1.18e-20 | 5.37e-21 |
| CC | GO:1902554 | serine/threonine protein kinase complex | 12/48 | 88/19717 | 1.75e-18 | 1.02e-16 | 4.61e-17 |
| CC | GO:0005667 | transcription factor complex | 17/48 | 365/19717 | 6.12e-18 | 2.37e-16 | 1.07e-16 |
| CC | GO:1902911 | protein kinase complex | 12/48 | 109/19717 | 2.58e-17 | 7.48e-16 | 3.39e-16 |
| CC | GO:0017053 | transcriptional repressor complex | 11/48 | 84/19717 | 8.46e-17 | 1.96e-15 | 8.91e-16 |
| MF | GO:0016538 | cyclin-dependent protein serine/threonine kinase regulator activity | 9/48 | 49/17697 | 6.80e-15 | 9.51e-13 | 4.08e-13 |
| MF | GO:0042826 | histone deacetylase binding | 9/48 | 111/17697 | 1.48e-11 | 1.03e-09 | 4.43e-10 |
| MF | GO:0033613 | activating transcription factor binding | 8/48 | 85/17697 | 6.53e-11 | 3.05e-09 | 1.31e-09 |
| MF | GO:0030332 | cyclin binding | 6/48 | 30/17697 | 1.63e-10 | 4.56e-09 | 1.95e-09 |
| MF | GO:0097472 | cyclin-dependent protein kinase activity | 6/48 | 30/17697 | 1.63e-10 | 4.56e-09 | 1.95e-09 |
| KEGG | hsa04110 | Cell cycle | 26/42 | 124/8076 | 5.93e-38 | 4.87e-36 | 2.12e-36 |
| KEGG | hsa04218 | Cellular senescence | 23/42 | 156/8076 | 2.33e-29 | 9.55e-28 | 4.17e-28 |
| KEGG | hsa04151 | PI3K-Akt signaling pathway | 11/42 | 354/8076 | 1.2369E-06 | 4.6103E-06 | 2.0122E-06 |
| KEGG | hsa04310 | Wnt signaling pathway | 7/42 | 160/8076 | 1.5821E-05 | 4.3245E-05 | 1.8875E-05 |
| KEGG | hsa04330 | Notch signaling pathway | 5/42 | 53/8076 | 7.1048E-06 | 2.2407E-05 | 9.7799E-06 |
